# βA3/A1-crystallin is a critical mediator of STAT3 signaling in optic nerve astrocytes

**DOI:** 10.1038/srep08755

**Published:** 2015-03-04

**Authors:** Mallika Valapala, Malia Edwards, Stacey Hose, Jianfei Hu, Eric Wawrousek, Gerard A. Lutty, J. Samuel Zigler, Jr., Jiang Qian, Debasish Sinha

**Affiliations:** 1 Wilmer Eye Institute, The Johns Hopkins University School of Medicine, Baltimore, Maryland 21287, USA; 2 National Eye Institute, National Institutes of Health, Bethesda, Maryland 20892, USA

## Abstract

We have previously reported that in the Nuc1 rat, which has a spontaneous mutation in *Cryba1* (the gene encoding βA3/A1-crystallin), astrocytes exhibit decreased Notch signaling, leading to reduced promoter activity for glial fibrillary acidic protein (GFAP). Interestingly, in both Nuc1 astrocytes and in wild type astrocytes following knockdown of *Cryba1*, vascular endothelial growth factor (VEGF) secretion is decreased. This has led us to explore signaling mediators that could be regulated by βA3/A1-crystallin to modulate both GFAP and VEGF. Several studies have shown that the signal transducer and activator of transcription 3 (STAT3) is involved in the co-regulation of GFAP and VEGF. We show that STAT3 and βA3/A1-crystallin may co-regulate each other in astrocytes. Such co-regulation would create a positive feedback circuit; i.e., in the cytosol of astrocytes, βA3/A1-crystallin is necessary for the phosphorylation of STAT3, which then dimerizes and translocates to the nucleus to form DNA-binding complexes, activating transcription of *Cryba1*. This stoichiometric co-regulation of STAT3 and *Cryba1* could potentiate expression of GFAP and secretion of VEGF, both of which are essential for maintaining astrocyte and blood vessel homeostasis in the retina. Consistent with this idea, *Cryba1* knockout mice exhibit an abnormal astrocyte pattern and defective remodeling of retinal vessels.

The primary vascular layer in the retina is intimately associated with the underlying meshwork of astrocytes that emerge from the optic nerve^1^. Although, it has been suggested that the astrocyte template (the honeycomb-like arrangement of astrocytes in the developing retina) guides the endothelial cells to facilitate vessel growth^2^, the molecular mechanisms underlying this process remain elusive. Previous studies have shown that plasticity of blood vessels is closely associated with the interplay between astrocytes, endothelial cells and pericytes^3^,^4^. Indeed, retinal astrocytes are only found in species with vascularized retinas^5^. We have previously shown that Notch signaling is important for proper astrocyte template formation and for maintenance of vascular homeostasis in the retina^6^. Although several studies have suggested the importance of the Notch signaling pathway in the development and maintenance of the retinal vasculature^7^,^8^,^9^, our knowledge of the mechanisms by which the Notch pathway and its downstream effectors mediate vascular homeostasis in the retina is very limited.

Emerging evidence suggests dynamic interactions between Notch signaling and VEGF, an inducer of angiogenesis in multiple cell types^10^,^11^. Recent reports show that inhibition of Notch signaling suppresses endothelial tip cell formation by reducing the levels of VEGF^11^,^12^. During retinal vascular development, astrocyte-derived VEGF is believed to provide guidance cues to the growing endothelial tip cells; hence, astrocytes are important for the stabilization and maturation of blood vessels^13^. These results suggest that the Notch signaling pathway and its downstream mediators are involved in astrocyte template formation and the subsequent remodeling of retinal vessels. However, the signaling mediators linking Notch to VEGF expression in astrocytes are unknown. One such signaling intermediate could be STAT3, a member of the signal transducers and activators of transcription (STAT) family, latent gene regulating proteins that are translocated to the nucleus and regulate gene transcription^14^. Previous studies have shown a direct link between Notch signaling and STAT3 activation to be important for astroglial differentiation and regulation of GFAP expression^15^,^16^. It has been shown that Notch signaling induces astrocyte differentiation in gliogenic cells by STAT3-mediated activation of the GFAP promoter^17^. Thus, Notch and its downstream signaling mediators are involved in the regulation of GFAP, and this pathway is also important for the regulation of astrocyte-derived VEGF. This prompted us to determine if STAT3 activation might also be affected by βA3/A1-crystallin in astrocytes, as we have recently shown for Notch^6^.

We report here that inhibition of the Notch pathway decreases the level of astrocyte-derived VEGF both in Nuc1 astrocytes and in wild type (WT) cells upon knockdown of *Cryba1*. The levels of STAT3 phosphorylated at tyrosine 705 (Y705) were reduced significantly in astrocytes lacking functional βA3/A1-crystallin relative to WT. Phosphorylation of STAT3 leads to the formation of homodimers that translocate to the nucleus and are critical for STAT3 dependent transcriptional activation. To investigate whether STAT3 could regulate transcription of *Cryba1*, we took advantage of the ENCODE project^18^, in which genome-wide binding locations were experimentally determined for a large number of transcription factors using a ChIP-chip or ChIP-seq approach in HeLa and MCF10A cell lines. Synteny mapping of the human to mouse genome identified STAT3 binding sites in the mouse *Cryba1* gene, and using quantitative real-time PCR as well as a promoter-based luciferase assay, we confirmed that *Cryba1* possesses a functional STAT3 binding site in the promoter region. Moreover, inhibition of Notch with N-[N-(3, 5- difluorophenacetyl)-L-alanyl]-S-phenylglycine t-butyl ester (DAPT) reduced phospho-STAT3 in both WT cells and in astrocytes where *Cryba1* is non-functional. This suggests that in astrocytes, STAT3 and βA3/A1-crystallin are co-regulated. Such co-regulation leads to a positive feedback loop, with βA3/A1-crystallin participating in the phosphorylation of STAT3 in the cytosol and, in turn, STAT3 regulating the transcription of *Cryba1* in the nucleus. We therefore provide in this study mechanistic insights as to how βA3/A1-crystallin modulates the Notch/STAT3 signaling axis to regulate normal functions of astrocytes in the retina, including secretion of VEGF and expression of GFAP. Interestingly, *Cryba1* knockout mice also exhibit an abnormal astrocyte template and defective remodeling of the retinal vessels, suggesting that βA3/A1-crystallin is essential for normal functioning of astrocytes in the optic nerve and the retina.

## Results

### Inhibition of Notch signaling reduces secretion of VEGF in optic nerve astrocytes

**Figure 1:**
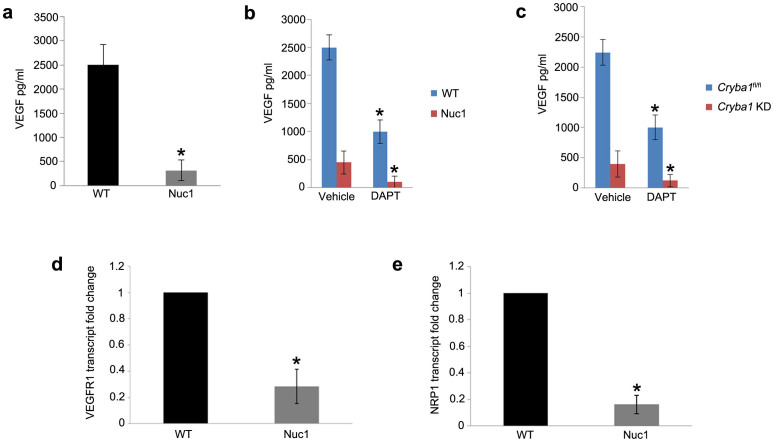
Impaired Notch signaling decreases VEGF secretion in wild type astrocytes and in astrocytes lacking functional βA3/A1-crystallin. (a). VEGF quantikine ELISA to detect levels of VEGF secreted into the medium showed significant reduction (~85%) in Nuc1 cells compared to WT cells. (b). Treatment with DAPT significantly reduced secreted levels of VEGF in both WT (~60%) and Nuc1 (~75%) astrocytes compared to the respective vehicle-treated controls (c). Adenoviral *Cre*-recombinase mediated knockdown of *Cryba1* in *Cryba1*^fl/fl^ mouse astrocytes (*Cryba1* KD*)* produced a 65% reduction in secretory VEGF and *Cryba1*^fl/fl^ control astrocytes showed ~55% reduction in VEGF level compared to vehicle-treated cells. (d). qRT-PCR analysis revealed ~70% reduction in the levels of VEGFR2 in Nuc1 astrocytes compared to the WT cells. e. Expression of NRP1 was reduced by ~80% in Nuc1 astrocytes compared to WT cells. Error bars indicate s.d.; *P<0.05.

Because of the abnormal retinal vasculature in animal models where βA3/A1-crystallin is non-functional in astrocytes^19^,^20^, we determined the levels of secreted VEGF in the conditioned medium of cultured WT and Nuc1 astrocytes. Astrocyte-derived VEGF is important for the stabilization and maturation of retinal vessels^13^. Recent evidence suggests potential dynamic interactions between the Notch signaling pathway and VEGF^11^. Our results show that the level of VEGF in the conditioned medium from Nuc1 astrocytes is reduced by ~85% compared to WT (Fig. 1, a). Next, we tested whether the Notch inhibitor, DAPT affects the secretion of VEGF. Our results show that DAPT significantly reduced levels of VEGF in the medium from both WT (~60%) and Nuc1 (~75%) astrocytes (Fig. 1, b). Mouse astrocytes from our previously generated *Cryba1* floxed mice (*Cryba1*^fl/fl^) were also used in this study^6^. Monolayer *Cryba1*^fl/fl^ astrocytes were infected either with control adenovirus (Ad CMV eGFP), or *Cre* recombinase adenovirus (Ad CMV *Cre*-RSV GFP) to knockdown *Cryba1* (*Cryba1* KD). *Cryba1* KD astrocytes exhibited a similar reduction in secretory VEGF as did *Cryba1*^fl/fl^ astrocytes (Fig. 1, c). These results suggest that the Notch pathway plays a key role in the secretion of VEGF, and that inhibition of this pathway in cells with non-functional βA3/A1-crystallin has a more pronounced effect on VEGF secretion. Our studies also show that rat optic nerve astrocytes express VEGF receptor 1 (VEGFR1) and the co-receptor Neuropilin-1 (NRP1), whose transcript levels are significantly downregulated in Nuc1 compared to WT astrocytes (Fig. 1, d and e).

### Astrocytes deficient in βA3/A1-crystallin protein exhibit altered levels of total and activated STAT3

**Figure 2:**
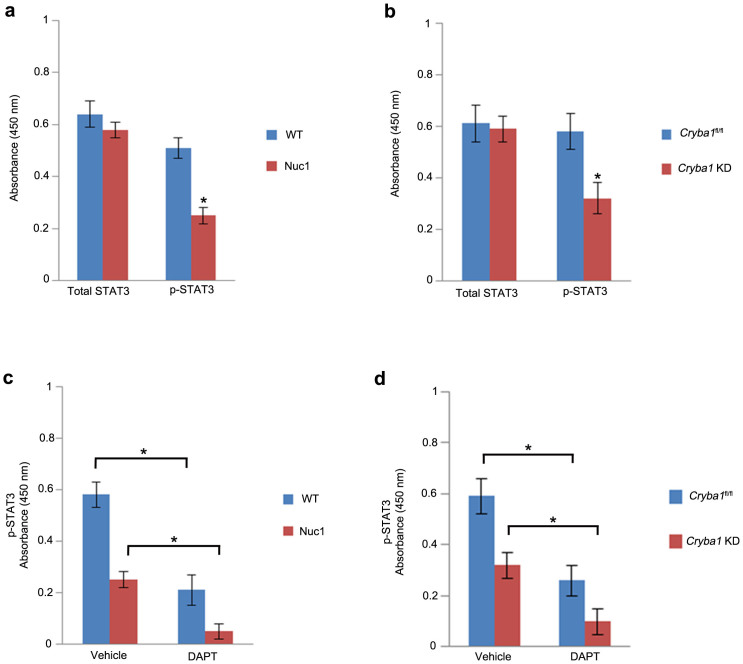
p-STAT-3 is impaired in astrocytes lacking βA3/A1-crystallin. (a). Analysis of WT and Nuc1 astrocyte lysates with total STAT3 and p-STAT3 antibodies using sandwich ELISA. No difference in the level of total STAT3 was observed between WT and Nuc1 astrocytes, whereas, the level of p-STAT3 was significantly reduced (~50%) in Nuc1 astrocytes compared to WT astrocytes. (b). *Cryba1* KD mouse astrocytes also showed significant reduction in the level of p-STAT3 compared to the *Cryba1*^fl/fl^ controls. Levels of total STAT-3 were unchanged. (c). Both WT (~57%) and Nuc1 (~75%) rat astrocytes treated with DAPT showed significant reduction in p-STAT3 compared to vehicle-treated cells. (d). Likewise, *Cryba1*^fl/fl^ and *Cryba1* KD astrocytes showed ~ 50% and ~62% reduction in p-STAT3 compared to respective vehicle-treated cells. Error bars indicate s.d.; *P<0.05.

We performed a sandwich ELISA assay using antibodies to total STAT3 and to STAT3 phosphorylated (p-STAT3) at tyrosine 705 (Y705) to quantitate protein levels in WT and Nuc1 astrocytes. Interestingly, no difference in the level of total STAT3 was observed when WT and Nuc1 astrocytes were compared; however, the level of p-STAT3 was reduced by ~50% in Nuc1 astrocytes relative to WT cells (Fig. 2, a). In *Cryba1* KD mouse astrocytes, we also observed reduced levels (~45%) of p-STAT3 compared to the *Cryba1*^fl/fl^ controls (Fig. 2, b). Levels of total STAT3 remained unchanged.

Previous studies have suggested that the Notch pathway is an important upstream regulator of STAT3 signaling and that it plays a crucial role in determining glial cell fate^15^,^16^. Since our previous studies have shown impaired Notch signaling in astrocytes lacking functional βA3/A1-crystallin^6^, we wanted to determine if inhibition of the Notch pathway directly affects STAT3 activation. We evaluated the effect of the Notch pathway inhibitor, DAPT, on the activation of STAT3. Our studies show that treatment with DAPT reduced p-STAT3 significantly in both WT (~57%) and Nuc1 rat astrocytes (~75%). Moreover, in the presence of DAPT, *Cryba1*^fl/fl^ and *Cryba1* KD astrocytes showed ~50% and ~62% reduction in p-STAT3 levels compared to the respective vehicle controls (Fig. 2, c and d). These results suggest that phosphorylation of endogenous STAT3 is impaired in astrocytes lacking functional βA3/A1-crystallin.

### βA3/A1-crystallin affects STAT3 activation in mouse optic nerve astrocytes

**Figure 3:**
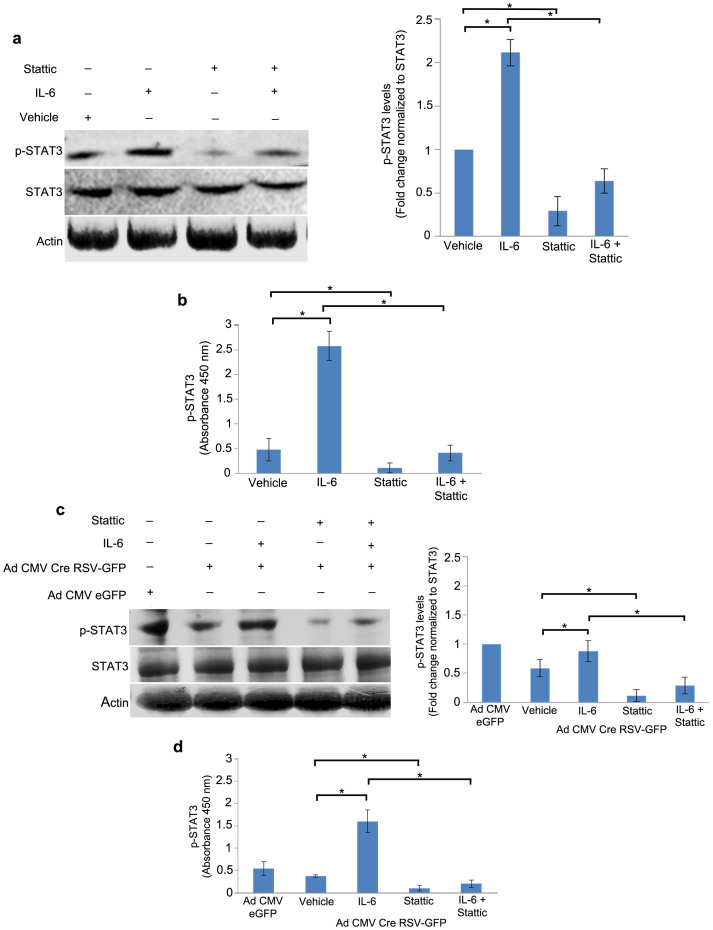
βA3/A1-crystallin modulates the phosphorylation of STAT3 in astrocytes. (a). Immunoblot analysis of total and p-STAT3 in WT astrocytes upon treatment with IL-6 and Stattic showed a 2.1 fold increase in the levels of p-STAT3 in astrocytes treated with IL-6 and ~70% reduction in p-STAT3 upon treatment with Stattic compared to vehicle-treated astrocytes. A ~70% reduction in p-STAT3 in astrocytes pre-treated with IL-6 upon treatment with Stattic compared to astrocytes treated with IL-6 alone was observed. (b). Analysis of p-STAT3 levels by ELISA revealed 5 fold induction of p-STAT3 in the presence of IL-6 and ~76% reduction in the presence of Stattic compared to the vehicle control. Stattic significantly decreased the levels of p-STAT3 in the presence of IL-6 (~85%) compared to the astrocytes treated with IL-6 alone. (c). However, in *Cryba1* KD astrocytes there was only modest activation (1.3 fold) of p-STAT3 in the presence of IL-6. Significant inhibition of p-STAT3 was observed in the presence of Stattic. (d). ELISA data revealed that IL-6 treatment induced p-STAT3 by 4.5 fold in *Cryba1* KD astrocytes compared to vehicle-treated astrocytes. Stattic decreased the level of p-STAT3 by ~80% when treated alone and by ~85% when treated in combination with IL-6 compared to vehicle-treated and IL-6 treated *Cryba1* KD astrocytes. Error bars indicate s.d.; *P<0.05.

The STAT family of transcription factors is known to be activated in response to cytokines and growth factors via phosphorylation at Y705, leading to subsequent dimerization and translocation to the nucleus^21^. Interleukin-6 (IL-6) binding to the gp130 receptor stimulates STAT3 phosphorylation by Janus activated kinase 2 (JAK2)^21^. The activity of STAT3 can also be inhibited by several pharmacological inhibitors. Recently, Stattic, a small molecule inhibitor of STAT3, has been developed that selectively inhibits the function of the STAT3 SH2 domain, thereby preventing phosphorylation and activation of STAT3^22^. In several cell types, Stattic has been shown to selectively inhibit activation, dimerization and nuclear translocation of STAT3^23^,^24^. In our studies, we used IL-6 and Stattic to activate and inhibit STAT3 phosphorylation, respectively, *in* *vitro*. Immunoblot analysis revealed a 2.1 fold increase in the levels of p-STAT3 in astrocytes treated with IL-6 and ~70% reduction in astrocytes treated with Stattic compared to vehicle-treated cells. Stattic also effectively reduced p-STAT3 levels in cells treated with IL-6 by ~70%, when compared to cells treated with IL-6 alone (Fig. 3, a) We also show by ELISA that IL-6 treatment increased the levels of p-STAT3 protein by 5 fold, while treatment with Stattic decreased p-STAT3 by ~76%, compared to the vehicle control. Stattic also decreased the levels of p-STAT3 in the presence of IL-6 by ~85% compared to the cells treated with IL-6 alone (Fig. 3, b). *Cryba1* KD mouse astrocytes showed only modest increase (1.3 fold) in p-STAT3 in the presence of IL-6 compared to vehicle-treated cells. However, Stattic either alone or in the presence of IL-6 decreased the levels of p-STAT3 in *Cryba1* KD astrocytes (Fig. 3, c). Similarly, ELISA data show that IL-6 treatment induced p-STAT3 by 4.5 fold in *Cryba1* KD astrocytes compared to vehicle-treated cells. Moreover, in *Cryba1* KD astrocytes, Stattic decreased the levels of p-STAT3 by ~80% when treated alone and ~85% when treated in combination with IL-6 compared to vehicle-treated and IL-6 treated *Cryba1* KD astrocytes, respectively (Fig. 3, d). These results suggest that βA3/A1-crystallin is an important molecule necessary for the phosphorylation of STAT3 in the cytosol of optic nerve astrocytes.

### The expression of *Cryba1* in optic nerve astrocytes is regulated by STAT3

**Figure 4:**
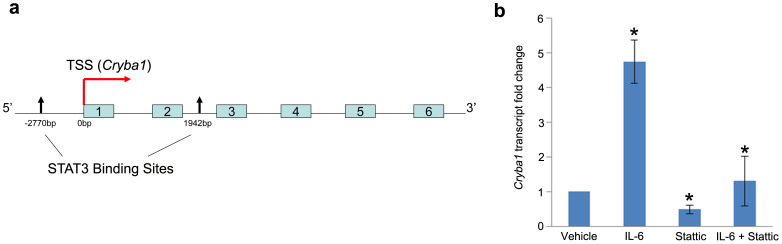
STAT3 regulates the expression of *Cryba1* in astrocytes. (a). Schematic diagram of the mouse *Cryba1* gene showing the transcription start site (TSS) and the two STAT3 binding sites, one 2750 bp upstream of the start site (promoter region) and the other 1942 bp from the TSS in Intron 2. Exons are shown in boxes. (b). Treatment of WT astrocytes with IL-6 resulted in 4.5 fold increase in the expression of *Cryba1* and Stattic treatment decreased the expression of *Cryba1* by ~51% compared to vehicle treated astrocytes. In astrocytes treated with both IL-6 and Stattic the expression of *Cryba1* was reduced by ~73% compared to astrocytes treated with IL-6 alone. Error bar indicate s.d.; *P<0.05.

The ENCODE program^18^ suggested that there are two potential STAT3 binding sites in the *Cryba1* gene, one in the promoter and another within intron 2 (Fig. 4, a). We therefore hypothesized that STAT3 might regulate the expression of *Cryba1* and performed quantitative real time PCR (qRT-PCR) on astrocytes treated with IL-6 and Stattic. Our data suggest that IL-6 treatment of WT rat astrocytes results in a 4.5 fold increase in the expression of *Cryba1* and that Stattic treatment decreased the expression of *Cryba1* by ~51% compared to vehicle treated astrocytes. In astrocytes treated with both IL-6 and Stattic, the expression of *Cryba1* was reduced by ~73% compared to cells treated with IL-6 alone (Fig. 4, b). Our results provide novel evidence that IL-6 increases, and Stattic decreases, expression of *Cryba1* in WT astrocytes, supporting our hypothesis that STAT3 regulates the expression of *Cryba1* in astrocytes.

**Figure 5:**
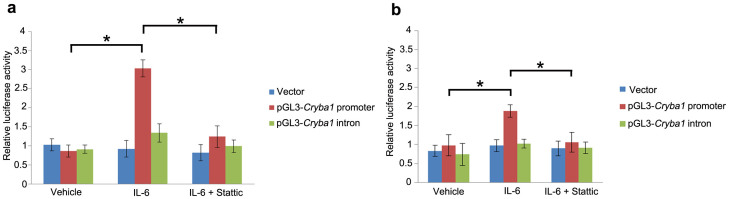
STAT3 binds specifically to *Cryba1* promoter *in vitro*. (a). A 3.2 fold induction of luciferase activity was observed in WT astrocytes transfected with pGL3-*Cryba1* promoter vector and treated with IL-6 compared to astrocytes treated with the vehicle control. WT astrocytes transfected with pGL3-*Cryba1* promoter vector and pre-treated with IL-6 in the presence of Stattic resulted in ~60% reduction in luciferase activity compared to astrocytes treated with IL-6 alone. (b). IL-6 treated Nuc1 astrocytes transfected with pGL3-*Cryba1* promoter vector also exhibited induction of luciferase activity (1.8 fold). Nuc1 astrocytes transfected with pGL3-*Cryba1* promoter and pre-treated with IL-6 resulted in ~44% reduction in luciferase activity in the presence of Stattic compared to IL-6 treated astrocytes. Treatment with IL-6 and/or Stattic had no effect in WT or Nuc1 astrocytes transfected with the intron vector. Error bars indicate s.d.; *P<0.05.

**Figure 6:**
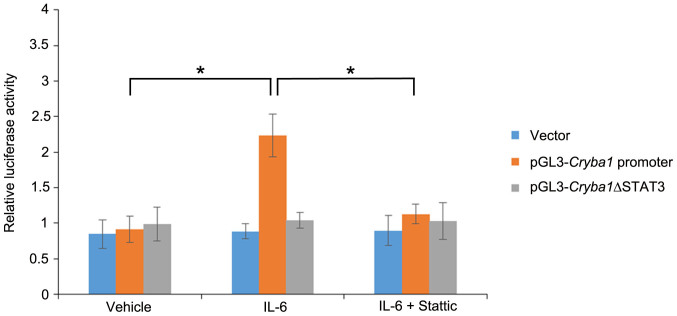
Active STAT3 binding site in the *Cryba1* promoter. A 2.5 fold induction of luciferase activity was observed in WT mouse astrocytes transfected with the pGL3-*Cryba1* promoter vector and treated with IL-6, compared to astrocytes treated with the vehicle control. Treatment with Stattic + IL-6 resulted in ~50% reduction in luciferase activity. In contrast, when WT astrocytes were transfected with the pGL3-*Cryba1*ΔSTAT3 vector, eliminating the STAT3 binding sites, luciferase activity was not affected by treatment with IL-6 or Stattic. Error bars indicate s.d.; *P<0.05.

In order to confirm that STAT3 binds at the sites in the promoter and intron 2 of the *Cryba1* mouse gene (Fig. 4, a), we performed luciferase assays. The predicted STAT3 binding site in the *Cryba1* promoter was cloned into the pGL3-promoter vector, upstream of the firefly luciferase reporter gene. The STAT3 binding site in intron 2 of the *Cryba1* gene was cloned downstream of the luciferase gene in the pGL3 vector. The pGL3 vector constructs containing the promoter and intron STAT3 binding sites were each co-transfected into WT and Nuc1 astrocytes along with the pRL-CMV vector carrying the Renilla luciferase gene under the control of the cytomegalovirus promoter. A significant induction (3.2 fold) of luciferase activity was observed in WT astrocytes transfected with pGL3-*Cryba1* promoter compared to the cells treated with vehicle. We did not detect any significant changes in luciferase activity in cells transfected with pGL3-*Cryba1* intron or with empty vector transfected cells. WT cells transfected with pGL3-*Cryba1* promoter and pre-treated with IL-6 in the presence of Stattic had ~60% reduction in luciferase activity compared to cells treated with IL-6 alone (Fig. 5, a). Furthermore, IL-6 treatment of Nuc1 astrocytes transfected with pGL3-*Cryba1* promoter resulted in 1.8 fold induction of luciferase activity. Luciferase activity was not induced in Nuc1 astrocytes transfected with pGL3-*Cryba1* intron or with empty vector. Nuc1 astrocytes transfected with pGL3-*Cryba1* promoter and pre-treated with IL-6 had a ~44% reduction in luciferase activity in the presence of Stattic compared to cells treated with IL-6 alone (Fig. 5, b). In addition, to further validate the existence of a functional STAT3 binding site in the *Cryba1* promoter, we generated a *Cryba1* STAT3 deletion construct (pGL3-*Cryba*1ΔSTAT3) by deleting the potential STAT3 binding site in the *Cryba1* promoter. As noted above (Fig. 5, a), a significant induction of luciferase activity was observed when astrocytes were transfected with pGL3-*Cryba1* promoter vector in the presence of IL-6. However, in astrocytes transfected with the pGL3-*Cryba1*ΔSTAT3 construct, luciferase activity was close to baseline levels in the presence of IL-6. To confirm that the activity was specific to STAT3, we treated the cells with Stattic in addition to IL-6. Stattic inhibited the luciferase activity only in astrocytes transfected with the pGL3-*Cryba1* promoter vector (Fig. 6). These results confirm the presence of an active STAT3 binding site in the region 2774 to 2756 base pairs upstream of the transcription start site in the *Cryba1* promoter (Supplementary Fig. 1).

### *Cryba1* knockout mice show abnormal retinal astrocytes and remodeling of the blood vessels

**Figure 7:**
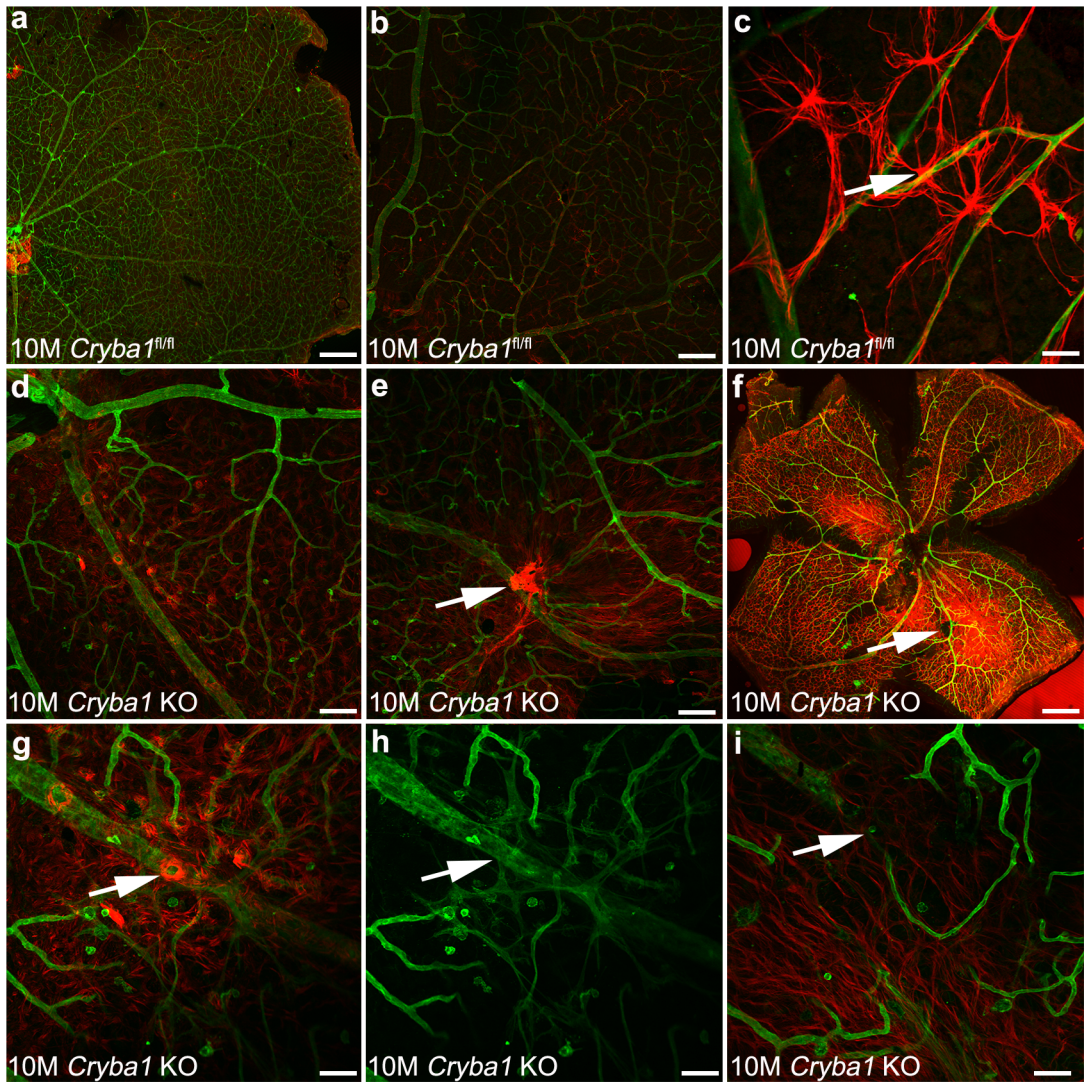
*Cryba1* knockout mice exhibit an abnormal astrocyte template and retinal vessels. Retinal flat mounts from 10 month old *Cryba1*^fl/fl^ (controls) or *Cryba1* KO mice were stained with GFAP (red; astrocytes) and GS isolectin (green; blood vessels). Control retina showing normal astrocyte/blood vessel patterning (a, b, c). (c) Higher magnification demonstrated the astrocyte endfeet on retinal vessels (arrow). The *Cryba1* KO retinas have abnormal astrocyte patterning (d–f) with astrocyte aggregates in focal areas (arrows in e and f). In some areas, astrocytes form swirls on retinal vessels (arrow in g). The retinal vessels of the *Cryba1* KO mice show unusual budding and networks (arrow in h) as well as constriction of larger vessels with abnormal orientation (arrow in i). n = 5 *Cryba1*^fl/fl^ animals and 5 *Cryba1* KO animals. Bar = 500,000 nm (f), 100 µm (a, b, d), and 50 µm (c, e, g, h, i).

We generated *Cryba1* knockout (KO) mice to further validate that loss of *Cryba1* affects astrocyte patterning as well as subsequent remodeling of the retinal vessels *in vivo*. *Cryba1* floxed mice were crossed with BEST1 (Bestrophin-1)-*cre* mice to generate RPE-specific conditional knockout mice^25^. However, it is known that germline deletion of floxed alleles may occur when floxed mice are maintained for multiple generations with the BEST1-*cre* allele, creating a global knockout of the floxed gene^26^. We used this as a strategy to generate *Cryba1* complete KO mice. In contrast to the Nuc1 rat, the lens is not ruptured in the *Cryba1* KO mice. Retinal flat mounts from 10 month old *Cryba1*^fl/fl^ or *Cryba1* KO mice were stained with GFAP (red; astrocytes) and GS isolectin (green; blood vessels). The *Cryba1*^fl/fl^ retinas showed the expected close association of retinal vessels and astrocytes (Fig. 7a–c). Astrocytes were observed in an organized honeycomb-like pattern across the entire retina (Fig. 7 b, c). Upon closer investigation, the astrocyte endfeet were clearly visible on retinal vessels (Fig. 7, c). In contrast, *Cryba1* KO retina showed an abnormal pattern (Fig. 7d–f), with an apparent increase in the density of astrocytes in focal areas (Fig. 7 e, f). While a few astrocyte endfeet were observed on retinal vessels in *Cryba1* KO mice, they were mostly replaced by astrocyte bundles on vessels (Fig. 7, g). Vessels in these areas had unusual budding and pattern (Fig. 7, h). In some areas, large vessels were constricted with disrupted orientation (Fig. 7, i). Together, these data demonstrate that βA3/A1-crystallin is essential for normal astrocyte function in the retina and that loss of βA3/A1-crystallin in astrocytes can affect the normal remodeling of retinal vessels.

## Discussion

Astrocytes are critical for the proper functioning of the retina. During retinal histogenesis, they migrate from the optic nerve into the inner retina and are closely associated with retinal blood vessels^5^,^27^,^28^. Several studies have shown that astrocytes and retinal blood vessels are developmentally linked^29^. The basic molecular mechanisms of Notch signaling, and its importance in retinal vascular development, are becoming increasingly clear^7^,^12^,^30^,^31^. Still, a clear gap exists in the literature regarding our knowledge of the cellular and molecular interactions of astrocytes during vascular remodeling in the retina. Our previous studies using genetically engineered animal models and *in vitro* systems suggested that βA3/A1-crystallin, a lens structural protein and a member of the β/γ-crystallin superfamily, is involved in astrocyte template formation and remodeling of the retinal vessels^19^,^20^. We have also shown previously that βA3/A1-crystallin is localized to the lysosomes of astrocytes^6^ and retinal pigmented epithelium^25^, where it modulates the activity of the lysosomal proton pump vacuolar ATPase (V-ATPase). This novel function of βA3/A1-crystallin, mediated through Notch signaling in the endolysosomal system, is required for proper patterning of the honeycomb-like astrocyte template in the retina^6^. To further delineate the mechanisms by which βA3/A1-crystallin affects astrocyte template formation and to link that process to vascular remodeling of the retina, we focused on the signaling pathways and cellular processes possibly modulated by βA3/A1-crystallin. The factors in the signaling pathway linking Notch to VEGF expression in astrocytes are unknown. JAK/STAT and Notch signaling act synergistically to promote astrogenesis^32^. In this study, we provide novel evidence that STAT3 affects *Cryba1* expression in astrocytes, which in turn regulates proper functioning of the Notch signaling pathway and its downstream activation of STAT3. The pool of βA3/A1-crystallin in the cytosol regulates the Notch signaling pathway leading to the phosphorylation and activation of STAT3. While βA3/A1-crystallin is entirely cytoplasmic in lens fiber cells, the protein appears to be present in the cell nucleus as well as in the cytoplasm of astrocytes^19^. Thus, our data provide direct evidence that in astrocytes βA3/A1-crystallin is involved in a positive feedback loop with STAT3, which regulates signaling events essential to the maintenance of cellular homeostasis in the astrocytes.

Previous studies have suggested that astrocytes secrete factors essential for vascular remodeling, including VEGF^13^,^33^. Our data suggest that βA3/A1-crystallin regulates the secretion of VEGF via the Notch signaling pathway in astrocytes. In *Cryba1* KO mice, the retinal astrocytes are morphologically abnormal, and there is a striking difference in the organization of the astrocyte template. While vessels form in the *Cryba1* KO retina, the subsequent remodeling process required to provide a mature vascular network is deficient. The phenotype in the *Cryba1* KO mice and our previous studies on Notch signaling^6^, as well as the present study on STAT3 signaling, suggest that βA3/A1-crystallin is a regulatory protein in astrocytes that can coordinate interactions among intracellular signaling proteins. For example, we have previously shown that the loss of βA3/A1-crystallin in astrocytes leads to decreased Notch signaling^6^. Based on our data, we also postulated that Notch is involved in juxtacrine signaling in astrocytes. In Nuc1 astrocytes, defective juxtacrine Notch signaling may account for improper template formation and patterning by retinal astrocytes^19^. In the present study, loss of βA3/A1-crystallin affects phosphorylation of STAT3, which inhibits dimerization and translocation to the nucleus, thereby decreasing the binding of STAT3 to the *Cryba1* promoter to initiate transcription.

Thus, a positive feedback loop between βA3/A1-crystallin and STAT3 appears to be crucial for astrocyte patterning in the retina, as well as for subsequent remodeling of retinal vessels. Support for such an idea, is obvious in flat-mounts from *Cryba1* KO retina, which show abnormal astrocyte patterning, along with abnormal vascular branching and budding (Fig. 7). In addition, areas of astrocyte bundles were also observed with increased GFAP staining. The increased GFAP staining probably results from activation of Müller cells, as would be expected in response to retinal degenerative changes due to abnormal blood vessel remodeling. Müller cell processes were evident in these focal areas with clusters of astrocytes. We have previously reported that astrocytes in the Nuc1 rat, exhibit reduced promoter activity for GFAP as well as decreased GFAP protein expression. We speculate that the increased GFAP staining in focal areas of the *Cryba1* KO retina is the result of accumulation of both astrocytes and Müller cell processes. It is conceivable that the bundle-like astrocyte aggregates in the *Cryba1* KO mice reflect a survival strategy similar to that exhibited by Nuc1 astrocytes in culture when subjected to anoikis-potentiating conditions^34^. We have suggested previously that a subset of Müller cells may be controlled by an anoikis-mediated cell death process^34^. Further, we have shown that loss of βA3/A1-crystallin induces IGF-II and increases cell survival by regulating the PI3K/AKT/mTOR and ERK pathways, thereby protecting astrocytes from anoikis-mediated cell death^34^. Therefore, βA3/A1-crystallin appears to modulate various intracellular signaling proteins along different signaling pathways, possibly in a juxtacrine manner amongst astrocytes to maintain cellular homeostasis. Together, these studies demonstrate that βA3/A1-crystallin is essential for normal astrocyte function in the retina and that its loss may lead to retinal vascular pathologies^19^,^20^.

In summary, our data provide novel evidence that βA3/A1-crystallin is a local mediator in astrocytes, modulating the Notch/STAT3 signaling axis, affecting patterning of the astrocyte template, and stimulating the secretion of VEGF required for remodeling of retinal vessels. Such studies provide important insights into the critical interplay between astrocytes and retinal vascular cells, potentially identifying therapeutic targets, such as βA3/A1-crystallin^35^, for the treatment of some retinal vascular diseases.

## Methods

### Antibodies

The following antibodies were used in this study: STAT3 (Cell Signaling Technologies, 9139), p-STAT3 (Cell Signaling Technologies, 9145) and GFAP (Dako, ZO334). Goat anti-rabbit Cy3 (Jackson Immunoresearch, 111-165-003) was used as the secondary antibody. GS isolectin conjugated with Alexa 488 (Life Technologies, L21415) was used to label blood vessels.

### Primary culture of optic nerve astrocytes

Optic nerve astrocytes from P2 WT and Nuc1 homozygous rats and *Cryba1* floxed mice were cultured in DMEM-F12 medium containing 10% FBS, as recently described^6^.

### Quantitative real time PCR

Total RNA was extracted from wild type and Nuc1 cells using the RNeasy Plus Mini Kit (Qiagen, Valencia, CA) following the manufacturer's instructions. 2 µg of total RNA was reverse-transcribed to cDNA in a 20 µl reaction volume using Superscript Reverse Transcription Kit (Invitrogen, Carlsbad, CA) as described^6^. PCR amplification was performed utilizing the 7500 PCR Fast Real-Time System (Applied Biosystems, Carlsbad, CA) and custom made TaqMan probes for *Cryba1* (Mm00501613_m1), VEGFR1 (Rn00570815_m1), VEGFR2 (Rn00564986_m1), NRP1 (Rn00686106_m1), HPRT(Rn01527840_m1) and GAPDH (Rn01775763_g1*). All data were analyzed with the ABI 7500 Real Time PCR system using the data assist software (Applied Biosystems), and the graphs were plotted using Microsoft Excel. All experiments were performed at least three times in triplicate. Data are represented as mean ± standard deviation of the mean (SD). For statistical analysis, Student's t-test was performed, and a p-value of <0.05 was considered statistically significant.

### Luciferase assay

For luciferase assays, lipofectamine 2000 (Invitrogen) was used to transfect cells with the pGL3 promoter vector containing the *Cryba1* promoter spanning 100 bp on either side of the STAT3 binding site located 2750 bp upstream of the promoter. The vector containing *Cryba1* promoter is mentioned as pGL3-*Cryba1* promoter. We also cloned 100 bp on either side of the STAT3 binding site at 1942 bp in intron 2; this vector is referred to as pGL3-*Cryba1* intron. To generate the *Cryba1* promoter deletion construct, the region containing −2774 to −2756 bp was deleted from the pGL3-*Cryba1* promoter vector using In-Fusion^R^ HD Cloning Kit (Clontech Laboratories Inc., Mountain View, CA) following the manufacturer's instructions. This vector is referred to as pGL3-*Cryba*1ΔSTAT3. The Notch luciferase reporter construct (2.3 µg) together with pRL-CMV (0.1 µg; Promega, Madison, WI) carrying the Renilla luciferase gene under the control of the cytomegalovirus promoter was co- transfected. Luciferase activity was measured using a luciferase assay system (Promega). The reporter activity was calculated by normalizing the firefly luciferase value with that of the Renilla luciferase control vector and expressed as relative luciferase units. All data were obtained from at least three independent experiments. The relative promoter activities were depicted as the mean ± S.D.

### SDS-PAGE and Western blot analysis

SDS-PAGE and Western blot analysis were performed as described previously^6^.

### ELISA

Total VEGF in the conditioned medium of cultured cells was measured using quantitative sandwich ELISA kit (R&D Systems) according to the manufacturer's instructions. Path scan ELISA kit (Cell Signaling Technologies) using STAT3 and p-STAT3 (Tyr 705) coated antibodies was according to the manufacturer's instructions. The data is represented as absorbance units at 450 nm from at least three independent experiments. Data are represented as mean ± standard deviation of the mean (SD).

### *Cryba1* knockout (KO) mice

*Cryba1* floxed homozygous mice (*Cryba1*^fl/fl^) were generated as described previously^6^. *Cryba1*^fl/fl^ mice were mated with Best1-*cre* mice^26^ that express *Cre* recombinase specifically in RPE to generate *Cryba1* conditional knockout mice^25^. It is known that germline deletion of floxed alleles may occur when floxed mice are maintained for multiple generations with the BEST1-*cre* allele, creating a global knockout of the floxed gene^26^. We used this as a strategy to generate *Cryba1* complete knockout (KO) mice. All animal studies were conducted in accordance with the Guide for the Care and Use of Animals (National Academy Press) and were approved by the Animal Care and Use Committee of Johns Hopkins University.

### Retinal flat mounts and Confocal microscopy

Eyes to be used for flat mounts were fixed and prepared for confocal microscopy to analyze the astrocytes and retinal blood vessels as previously described^20^. GS isolectin conjugated with Alexa 488 (Invitrogen) was use to label blood vessels.

### Statistical Analysis

Statistical analysis was performed using Microsoft Excel. The P-values were determined by two-tail Student's t-test in a triplicate experiment representative of at least three independent experiments. Significance was defined as *P<0.05. Results are presented as mean ± S.D.

## Author Contributions

D.S. designed the study. M.V., M.E. and J.H. conducted the experiments. M.V., M.E., G.L., S.Z., J.Q. and D.S. analyzed the data. S.H. constructed figures for the manuscript and contributed to some experiments. E.F.W., S.Z. and D.S. participated in the generation of *Cryba1*-floxed and knockout mice. M.V., S.Z. and D.S. wrote the paper. All authors have approved the final manuscript.

## Supplementary Material

Supplementary Information Supplementary Figure
