# Supplementary material for: βA3/A1-crystallin is a critical mediator of STAT3 signaling in optic nerve astrocytes
Source: Sci Rep. 2015 Mar 4;5:8755. doi: 10.1038/srep08755 (PMC4348628; doi:10.1038/srep08755)
Supplement: Supplementary Information — Supplementary Figure [file srep08755-s1.pdf]

βA3/A1-crystallin is a critical mediator of STAT3 signaling in optic nerve astrocytes

Mallika Valapala[1], Malia Edwards[1], Stacey Hose[1], Jianfei Hu[1], Eric Wawrousek[2],

Gerard A. Lutty[1], J. Samuel Zigler, Jr.[1] Jiang Qian[1] and Debasish Sinha[1]*

[1]Wilmer Eye Institute, The Johns Hopkins University School of Medicine,

Baltimore, Maryland 21287, USA,

[2]National Eye Institute, National Institutes of Health, Bethesda, Maryland 20892 , USA.

*Corresponding Author:

Debasish Sinha

The Wilmer Eye Institute

The Johns Hopkins University School of Medicine

Baltimore, MD 21287

410-502-2100 (tel)

410-614-6728 (fax)

Debasish@jhmi.edu

-2800 -2770

CAGTTTCTCCCTTCATTTCTACTCCCTTGCCCTAAATTCCCTGAAAAGAGGTGGGACTAGGAGCTGGAGCTTTGAGGTAAGGTCCATGTAGGACAGGCAA
AGCAGAATCTCAGATAACAAGGGAAGTTCCACATCTTTGTGAAGAGACGTGGCTCAGGGCTAGAGTGTTCGTCTAACATGTGCAGGCCCTGGGTTGATTC
GCAGTGTCATGAAGGAAGCAACAGCCACTCAAGCCCCCATATCAGGCTATCCTTTTGATAATTAGCACAGGAGACCTCAGAATCCTCAAGTCTCTGGGAC
GTAAGGTGATAAAAGAAAATGATGTGTGTGTTGGGGGTGGGGCTGGCAGCTTAGGAATTCTTGGTGGACATATGCCAATGGTTTGCACACTCTCTGAGCC
ACACACAATCCCCCATGCAATGATTTTGTGCTTCTATTTCCTCTCTGAAATGAAAAGTTAACTAAATCAGTCTTTACTGAGCCAACAATTCATTCAAGTA
TAAAACTCTCCCCAAAGATCCTGAGTGGAAAAAAGAGTCCCAGGCAGTGCAGGTGGGATGGGCAGCTGTGTACAGCAGGGTGACAGGGCAGTGAAATTGT
CACATGTTCACTGTGCCTCTTCCAAAGTCACAAGACTCATTCATTTTCTCAAAATGGATTTATCAAGCTAAATAGAAGAAGCAGATAATTAGGTGGTATT
TATTTTTCAAAAATAACTGTTTTAATATATATAGTTTTCCCAGACAGGGTTTCTCTGTGTAGCCCTGGCTGTCCTAGAACTCACTCTGTAGACCAGGGTG
ATCATGAACTCACATGCCCAACTCTGCCTGGAATTAAAGGTCATCACTGCCTGGCTAACATAAAATGTATTCTTGTTGCTTAAAGTTCAAAGACTACAGA
AAAATATATTGTGAAAAAATTTGCCCTAAATCAAGTGGCTCAGTTCCCTTCTTTGAATCCCAATATCTCTAGTTTCTAAAGAAATTTTTACAAGGTGAGG
CCATACAAGTCAGTCCAGAGATTAGGAGATGAAAGAAGTAGTTGACCAAAGAAGTATTTTTCTTTTTTCTTTTTTGGTTTTTCAAGACAGGGTTTCTCTG
TGTAGCCTTGGCTATCCTGGAACTCACTCTGTAGACCAGGCTGTCCTTGAACTCAGAAATCCCCCTGCCTCTGCCTCCCAAGTGCTGGGATTAAAGGCAT
GCACCACCACACCCAGCTTATGTTTAATTTTTTTAATTAAATATTTTCTATATTTACATTTCAAATGTTATCCGCTTTCCTGGTTTCCCCTTCGAAAATC
CCCTCCCCCTGCTCCCCAACCTTCCCACTCCCATTCCTGGCCCTGGCCTTCCCCTATACTGGGGCATAGAACCTTCACAGGACCTGGGGCCTCGCCTCCC
ATTGATGACCGAAGAGGCCATCAAGAAGTATTTTTCTAAGATGGCAGTCTGGAAAGGAGAAAAAGGACTAAGACAGGGGGTGGGAAGTGGGGGACAGCAA
AATGGCTCAGCTGGTAAAGATGTTCCCTTCCACCACTGAATTTAATCCCTGGAACCCACTTGGTGGAAGGAGAGAACTGACTCTCAGAAACACACATGCA
CACACACGCACACACACACACACACACACACACACACACACACGCGCACACACGCACACACACACGCGCGCGCACACACACTCACACAAACTAAGT
AAGCAAATGTAAATGCACACACACACAAGTGATAACCACCAAAAAATCCCTCAGGTTATTAGCCCCAAATTTTTAAAAGCAAACTACAGTATTAAAGTGA
GTATTTAATAAAACAAAGTGAGATTCAGGTCTACCTGTCAATTGTAATTGAATTTAGCTACAGATGTGTAGAGAATAATCGAATAAGAAGCTTTGCTGGG
CAGTGGTGGCACACGCCTTTAATCTCAGCGCTCGGGAGACAGAGGCAGGCAGATTTCTGAGTTCTAGGCCAGCCTGGTCTTCAGAGTGAGTTCCAGGACA
GCCAGGGCTACACAGAGAAACCCTGTCTCGAAAAATCAAAACAAAGCAAAACAAAACAAAACCAAACCAAACCATGGGCTGGAGAGATGGCTCAGTGGGT
AAGAGCACTTACTGCTCTTCCGAAGGCCCTGAGTTCAAATCCCAGCAACCACATGGTGGCTTACAACCCTCCGTAATGAGATCTAATGCCCTCTTCTGAT
GCATCTGAAGTCAGCTACAGTATACTTATGCATAATAATAAAACTTTTGTTTGTTTTATTTTATTTTGTTTGTTTTGTTTTTGAGACAGGGTCTCATTAT
ATAGCCTGGGCTGTCAAAGAACTTAATATATAGATCAGCTGGCCCTGAAATCATAGAGATCCAACTCTGCCTCCGTCTCCTGAGTGCTGGGATCAAAGTC
TTGCCCCATGACTACTTCTGGCTCTGTGTGTGTGTGTGTGTGTGTGTGTGTGTGTGTGTGTGTGTGTGTGTGTGTGTGTGTGTGTGTGTACATATGCAGATGGT
CCATTTTGATGTGCTTTCCACAACCTTTCAACATTTTAGGTAGGTCCCAGGTGGAACAGGATAGGGACAAGCATTCACTGTAGAGCACTTCTGTCCTTAG
CTCCCAGCTGCTCTGAGAAGCCCAACAGTAGGGCCCCTGCACTTCTGGAACCCTAAACAAAGCTCTGAGGGACACCTCCCCCAATAGGCTGAGCCACTAA
AGCTCTGGGGATCAGGAGGAACAAAGAGAGAGGGAAAAGAGAGTGCTGACAGAGCCGCTGGCCATCATCTGTGGGACTATAAAGAGGGGATCCGGAGACG
AAGCTGCACCAGATGGAGACCCAGACTGTGCAGCGGGAGTTGGGTGAGTAAGGTCTTGACGGCGCCTTTATCTTTCCTTTCCTCGATAGACAGATGCAGA
GGAAAAACCAGTTGTGTCCCATTTAGAGGTTAGAATATTTGAAGGTAGAGAAAGTGGGGGACAGCTTCATTTGCTACCTTAAGTAGTTGCCTGTGGCAGT

STAT3 binding site   Transcription start site   Translation start site

Supplementary Figure: The nucleotide sequence of the mouse *Cryba1* promoter showing the putative STAT3 binding site (red). The ATG initiation codon is shown with a blue arrow and the transcriptional start site in green.
